# Nicotinamide riboside supplementation corrects deficits in oxytocin, sociability and anxiety of CD157 mutants in a mouse model of autism spectrum disorder

**DOI:** 10.1038/s41598-019-57236-7

**Published:** 2020-06-22

**Authors:** Maria Gerasimenko, Stanislav M. Cherepanov, Kazumi Furuhara, Olga Lopatina, Alla B. Salmina, Anna A. Shabalova, Chiharu Tsuji, Shigeru Yokoyama, Katsuhiko Ishihara, Charles Brenner, Haruhiro Higashida

**Affiliations:** 1 Department of Basic Research on Social Recognition and Memory, Research Center for Child Mental Development, Kanazawa University, Kanazawa, 920-8640 Japan; 2 Department of Socioneurosciences, United Graduate School of Child Development, Osaka University, Kanazawa University, Hamamatsu University School of Medicine, Chiba University, and University of Fukui, Kanazawa Campus, Kanazawa, 920-8640 Japan; 3 Laboratory for Social Brain Studies, Research Institute of Molecular Medicine and Pathobiochemistry, and Department of Biochemistry, Krasnoyarsk State Medical University named after Prof. V. F. Voino-Yasenetsky, Krasnoyarsk, 660022 Russia; 4 Department of Immunology and Molecular Genetics, Kawasaki Medical School, Kurashiki, Okayama 701-0192 Japan; 5 Department of Biochemistry, Carver College of Medicine, University of Iowa, Iowa City, IA 52242 USA

**Keywords:** Social behaviour, Autism spectrum disorders

## Abstract

Oxytocin (OT) is a critical molecule for social recognition and memory that mediates social and emotional behaviours. In addition, OT acts as an anxiolytic factor and is released during stress. Based on the activity of CD38 as an enzyme that produces the calcium-mobilizing second messenger cyclic ADP-ribose (cADPR), CD157, a sister protein of CD38, has been considered a candidate mediator for the production and release of OT and its social engagement and anti-anxiety functions. However, the limited expression of CD157 in the adult mouse brain undermined confidence that CD157 is an authentic and/or actionable molecular participant in OT-dependent social behaviour. Here, we show that CD157 knockout mice have low levels of circulating OT in cerebrospinal fluid, which can be corrected by the oral administration of nicotinamide riboside, a recently discovered vitamin precursor of nicotinamide adenine dinucleotide (NAD). NAD is the substrate for the CD157- and CD38-dependent production of cADPR. Nicotinamide riboside corrects social deficits and fearful and anxiety-like behaviours in CD157 knockout males. These results suggest that elevating NAD levels with nicotinamide riboside may allow animals with cADPR- and OT-forming deficits to overcome these deficits and function more normally.

## Introduction

Oxytocin (OT) plays a role in social recognition, behaviour, and memory through a positive feedback system involving OT-induced OT release in the brain^1–3^. OT is released in response to emotional, physical, and pharmacological stresses^1,2^. It is known that OT counteracts stress-induced anxiety. Accordingly, OT in the brain is considered an anxiolytic factor^2,3^.

CD38 and CD157 are two related cell-surface molecules that form the calcium-mobilizing second messenger, cyclic ADP-ribose (cADPR) from nicotinamide adenine dinucleotide (NAD)^4–8^, the central coenzyme of metabolism^9^. cADPR functions as a potential intracellular second messenger that triggers Ca^2+^ mobilization from ryanodine receptor Ca^2+^ pools to produce cellular responses^4,6^. In the hypothalamus, cADPR triggers an increase in intracellular free Ca^2+^ concentrations and, subsequently, Ca^2+^-dependent OT release from oxytocinergic neurons^10^. When this signalling cascade was blocked in CD38 knockout (CD38KO) mice, social memory and recognition and parental nurturing behaviours were disrupted, mainly due to reduced OT secretion^11,12^. The treated mice increased levels of social behaviour, which was invoked by local re-expression of human CD38 in the hypothalamus region or a simple subcutaneous supply of OT in CD38KO mice^11^. The phenotypes of CD157KO and CD38KO mice, in terms of social behaviour, are partly shared, but significant differences exist. CD157KO mice prominently display anxiety- and depression-like behaviours and social avoidance, but hyperactivity was not observed in CD38KO mice^8,13–15^.

Nicotinamide adenine dinucleotide (NAD) is synthesized by salvage of either of three forms of vitamin B3, nicotinic acid, nicotinamide, and nicotinamide riboside (NR) or from tryptophan in a *de novo* biosynthetic pathway^16–18^. Whereas nicotinic acid and tryptophan-dependent synthesis or tissue-restricted, all cells appear to synthesize nicotinamide phosphoribosyltransferase, and nicotinamide riboside kinase (NMRK)1, which confer the ability to utilize nicotinamide and NR, respectively^17^ In a number of conditions of metabolic stress, including heart failure^19^, noise-induced hearing loss^20^, central brain injury^21^ and peripheral neurodegeneration^22^, key metabolites such as NAD^+^ and/or NADPH^23^ are under attack. NR has unique properties of repleting the NAD metabolome in these conditions, largely due to the transcriptional induction of the NMRK1 and/or NMRK2 genes in conditions of metabolic stress. Postpartum constitutes an additional condition of metabolic stress that is accompanied by restribution of the NAD metabolome from the maternal liver to the mammary, where it drives lactation-associated biosynetic programs^24,25^. NR itself is a natural product that is found in milk^16,25,26^, which is orally available to people as an NAD-boosting vitamin^27,28^.

To test whether NR supplementation might address phenotypes associated with a model of an autism spectrum disorder, we chose to investigate male CD157KO mice. These mice have an intact CD38 gene and low circulating OT. In this genotype, males present a more scorable behavioural phenotype than do females including depression-like behaviour^13,14^ and anxiety-like beahviour with respect to the open arms of the elevated plus maze^13^. Here we show that biochemical and behavioural defecits of CD157KO mice are reversed by oral administration of NR.

## Results

### Phenotypes of CD157KO mice

**Figure 1:**
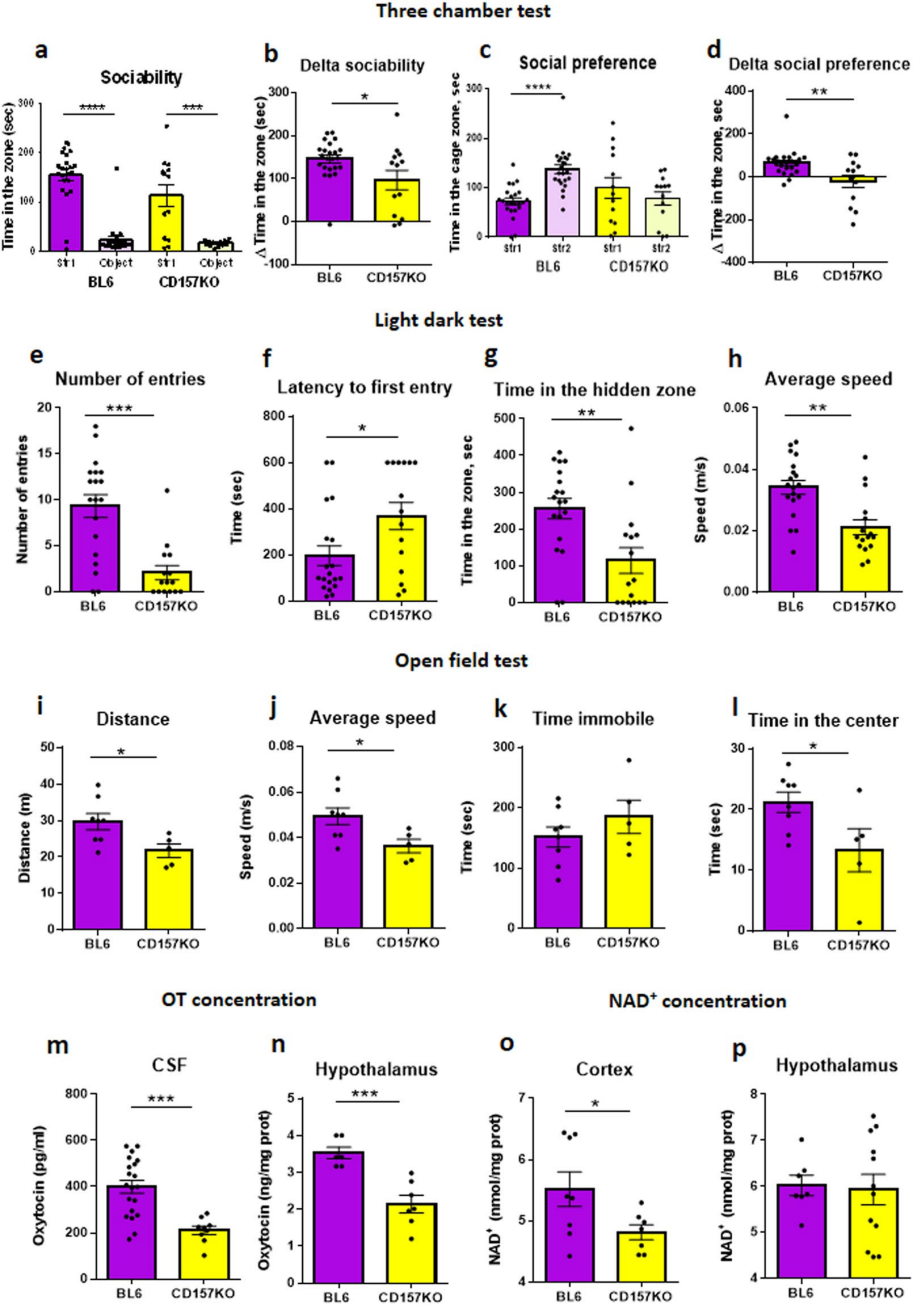
Social behaviour of adult wild-type (BL6) or CD157KO male mice. (**a–d**) Three chamber test. (**a**) The Sociability stage. Time spent around wire mesh cages with the social target (a conspecific male mouse, stranger 1, str 1) or with a non-social object (usually a plastic test tube, object). (**b**) Delta sociability (the time difference spent between the social and nonsocial targets calculated from (**a**)). (**c**) Social preference (time spent with the familiar mouse, str1, or with new mouse, str2. (**d**) Delta social preference (the time difference spent between the unfamiliar and familiar social targets calculated from (**c**)). (**e**–**h**) The light-dark transition test. (**e**) The number of entries from light to dark zone. (**f**) Latency to the first entry from the light to dark zones. (**g**) Time spent in the hidden zone. (**h**) The average speed in the light zone. (**i**–**l**) The open field test. (**i**) The distance traveled in all arenas (**i**), average speed (**j**), immobile time (**k**), and time spent in the center zone (**l**). Oxytocin concentrations in the cerebrospinal fluid (CSF, **m**) and the hypothalamus (**n**). NAD^+^ concentrations in the cortex (**o**) and hypothalamus (**p**). Unpaired *t*-test. *P < 0.05; **P < 0.01; ***P < 0.001; ****P < 0.0001.

Although there have been fragmentary reports about CD157KO mice^8,13,14,29,30^, here, we systematically summarized the reports on social interaction behaviour and concentrations of related molecules. Like humans, wild-type (C57BL6/N) and CD157KO mice are social creatures that prefer to spend time with another mouse than with an inanimate object (Fig. 1a,b)^31,32^. As shown in Fig. 1a, when placed in a box with an unfamiliar mouse (Stranger 1) in the left zone and an inanimate object in the right zone, wild-type males spent nearly all of their time with the mouse (unpaired *t*-test, *t*(44) = 10.13, *P* < 0.0001). CD157KO males had a somewhat more variable preference for the mouse but still showed a strong preference for social interaction than the object, which was confirmed by the time difference spent between the social and nonsocial targets (delta sociability; Fig. 1b; *t*(24) = 4.346. *P* < 0.001).

As shown in Fig. 1c, when the same test mice were reintroduced to the box with a second unfamiliar mouse (Stranger 2), wild-type mice spent approximately twice as much time with a new mouse than with the familiar one (Stranger 1; *t*(44) = 5.755, *P* < 0.0001). However, CD157KO mice displayed no preference for the new mouse (Fig. 1d; *t*(24) = 0.8542, *P* = 0.4015).

Mice are nocturnal and cautious by nature. When placed in the light side of a light/dark box, mice experience some discomfort and typically find their way to the dark side, where they feel less stressed^31^. Wild-type males behaved this way, as indicated by a number of different parameters, such as the number of entries into the dark side (Fig. 1e), latency to the first entry (Fig. 1f), time in the hidden zone (Fig. 1g) and average speed in the light zone (Fig. 1h). However, CD157KO mice showed fewer entries (Fig. 1e; *t*(32) = 4.646, *P* < 0.0001), had a longer latency to first entry into the dark zone (Fig. 1f; *t*(32) = 2.399, *P* < 0.05), spent less time in the hidden zone (Fig. 1g; *t*(32) = 3.596, *P* < 0.01) and had a lower average speed (Fig. 1h; *t*(32) = 4.165, *P* < 0.001).

The third test was the open field test for anxiety-like behaviour and locomotion activity^33^. Compared with wild-type mice, the distance moved (Fig. 1i; *t*(11) = 2.530, *P* < 0.05), average speed (Fig. 1j; *t*(11) = 2.504, *P* < 0.05), and time spent in the centre (Fig. 1l; *t*(11) = 2.283, *P* < 0.05) for CD157KO male mice were significantly lower than those of wild-type mice. However, there was no significant difference in immobile times (Fig. 1k; *t*(11) = 1.11, *P* = 0.2908).

Next, when we measured OT levels as a biomarker in the cerebrospinal fluid (CSF; Fig. 1m; *t*(27) = 4.297, *P* < 0.001) and hypothalamus (Fig. 1n; *t*(11) = 4.746, *P* < 0.001), the OT concentrations in CD157KO mice were significantly lower than those in wild-type mice.

The basal level of NAD^+^ was also measured in cortical and hypothalamic tissue (Fig. 1o,p) because the hypothalamus is the region in which OT is produced, and the cortex was used as the OT non-producing control region. CD157KO mice had a significantly lower NAD^+^ level in the cortex (*t*(13) = 1.196, *P* < 0.05) but not in the hypothalamus (*t*(17) = 0.1982, *P* = 0.8452).

The above associations between social behaviour and OT concentrations in CD157KO male mice are in good agreement with those in previously published reports^13,14,30^.

Behaviours in CD157KO female mice are illustrated in Supplementary Fig. 1. Untreated female mice of both genotypes displayed stronger levels of interest for social objects than nonsocial targets (wild-type female mice in Supplemental Fig. 1a Control; *t*(30) = 7.521, *P* = 0.0001; and CD157KO females in Supplemental Fig. 1b Control; *t*(22) = 3.300, *P* = 0.0033). However, unlike male mice, both wild-type and CD157KO female mice did not show a preference for novel social objects (Control in Supplementary Fig. 1c; *t*(30) = 1.287 *P* = 0.2078; Control in Supplementary Fig. 1d; *t*(22) = 1.840, *P* = 0.0794) These results indicate that CD157KO male mice, but not female mice, are useful models for psychiatric disorders with social behavioural impairments, because they display fewer social interactions, anxiety-like behaviours and/or social avoidance. Based on these characteristics of CD157KO male mice, we started to examine the effects of oral NR on social behavioural impairment. Adult wild-type and CD157KO male mice treated daily with either saline or 3–26 mg NR/mouse (approximately 100–1000 mg/kg of body weight) for 12 days did not exhibit any apparent changes, including movement dysfunction.

### NR corrects the social preference deficit of CD157KO males

**Figure 2:**
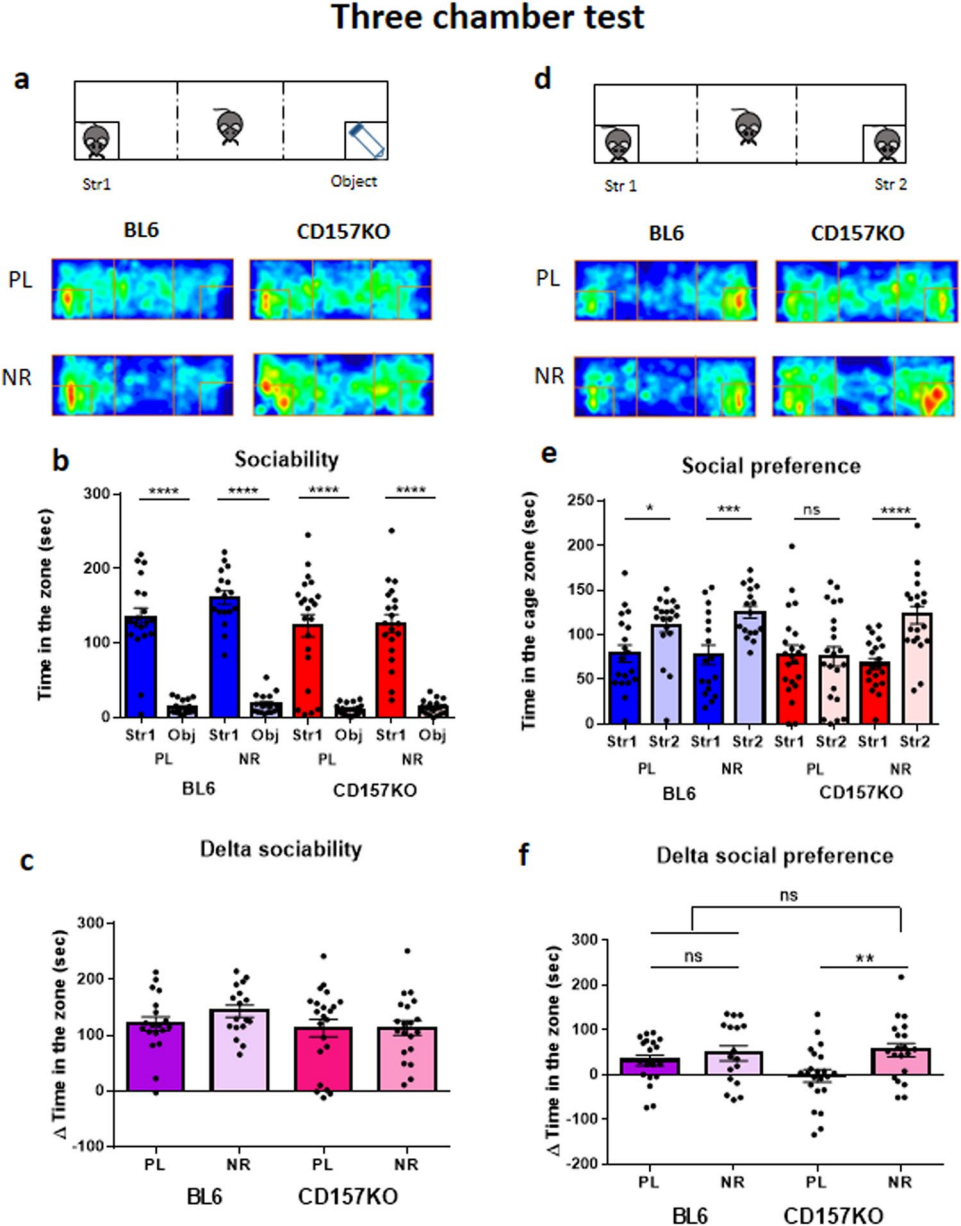
Social interaction of adult male wild-type (C57BL/6 N and BL6) and CD157KO mice in the three-chamber test. The sociability stage (**a**–**c**) and social preference stages (**d**–**f**). Experimental schemes in the sociability task when a stimulus male mouse (Str 1) was placed in the left chamber (**a**) or in the social novelty task when a new target male mouse (Str 2) was placed in the right chamber (**d**). Group occupancy plots of test mice initially place in the center in the sociability stage (**a**) or social preference (**d**) by the three-chamber box test after oral administration of saline (PL) or nicotinamide riboside (NR, 3 mg/mouse) treatment for 12 days. (**b**) Time spent in the wire mesh cage with the social target mouse (a conspecific male mouse, Str 1) or a non-social object (a test tube, Obj). Unpaired *t*-test shows significace between Str 1 and Obj at ****P < 0.0001. (**c**) The delta sociability represents time difference between social and non-social targets. (**e**) Time sp**e**nt by test wild-type (BL6) or CD157KO mice in the cage with a familiar (Str 1) or new unfamiliar (Str 2) target mouse is plotted. Note that social preference was observed in wild-type mice under 2 conditions (PL and NR). While no social preference was observed in CD157KO mice treated with saline (PL), preference was obvious in KO mice treated with NR. Unpaired *t*-test shows significance between Str 1 and Str 2 at *P < 0.05, **P < 0.01, ***P < 0.001, and ****P < 0.0001. (**f**) The time by NR-treated CD157 KO mice spent with an unfamiliar mice was significantly higher than that with a familiar mouse. Two-way ANOVA, effect of treatment, *F*_1,74_ = 6.412, P < 0.0135. Bonferroni’s *post hoc* analysis shows *P* < 0.01 between PL and NR.

We used three chamber tests to determine affinity for social targets (sociability) and new unfamiliar social targets compared with that for familiar targets (social preference). In control experiments with wild-type males, we performed daily gavage with either a saline placebo or 3 mg of NR for 12 days. These mice have a clear behavioural preference for a social target (Stranger 1) over a nonsocial target in sociability, which was not altered by NR (Figs. 2a,b; unpaired *t*-test, *t*(36) = 9.403, *P* < 0.0001 for placebo; *t*(32) = 14.98, *P* < 0.0001). Delta time, which was calculated by subtracting time in the non-social target zone from time in the social target zone, was essentially equal between genotypes and treatments (Fig. 2c; two-way ANOVA: effect of treatment, *F*_1,74_ = 0.7065, P = 0.4033; effect of genotype, *F*_1,74_ = 1.99, *P* = 0.1625; interaction, *F*_1,74_ = 0.6941, *P* = 0.4074).

CD157KO males had a somewhat more variable preference for the social target but still showed a strong preference for social interaction over the nonsocial object (Fig. 2a,b; *t*(42) = 7.449, *P* < 0.0001 for placebo; *t*(38) = 9.206, *P* < 0.0001 for NR). Because there was no sociability deficit in CD157KO males, it is reasonable to assume that there was no effect of either the placebo or NR.

As shown in Fig. 2d,e, CD157KO mice displayed no preference for interaction with the new (Stranger 2) mouse (*t*(42) = 0.1583, *P* = 0.8749). Remarkably, however, daily gavage of NR reversed this lack of preference for Stranger 2 to a level indistinguishable from that in wild-type males (Fig. 2e, *t*(38) = 4.689, *P* < 0.0001). Delta time (time spent in the two chambers, as defined above): Fig. 2f; two-way ANOVA: effect of treatment, *F*_1,74_ = 6.412, P < 0.0135, with *P* < 0.01 by Bonferroni’s *post hoc* analysis; effect of genotype, *F*_1,74_ = 0.9138, *P* = 0.3422; interaction between genotypes and treatment, *F*_1,74_ = 2.067, *P* = 0.1547). These data indicate that the brains of CD157KO males retained plasticity and were able to recognize and be interested in new mice when they were given a nutritional intervention.

### NR corrects the anxiety-related phenotype of CD157 males with respect to light/dark transitions

**Figure 3:**
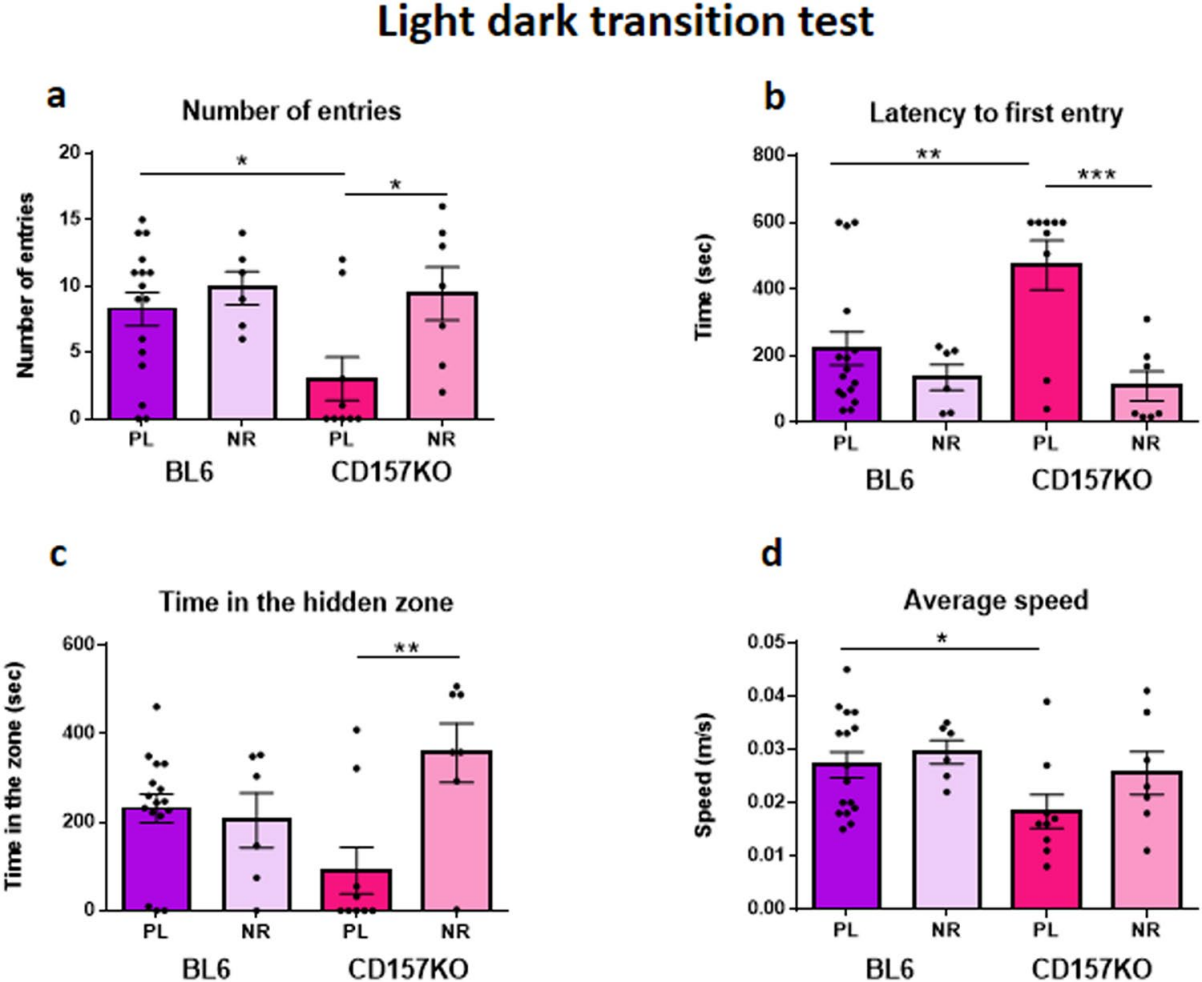
Effects of gavage supplementation of nicotinamide riboside (NR) for 12 days on anxiety-like behaviour in the light-dark transition test in wild-type (BL6) and CD157KO male mice, compared with that with saline treatment (PL). **(a)** Transition numbers between the two compartments. Two-way ANOVA; *F*_1,34_ = 5.766, *P* = 0.022; Bonferroni’s *post hoc* analysis revealed a significance between placebo and NR in CD157KO mice, P < 0.05. **(b)** Latency of the first entry into the dark zone. Two-way ANOVA; effect of treatment, *F*_1,34_ = 12.62, *P* = 0.0011; genotype and treatment interaction, *F*_1,34_ = 4.695, *P* = 0.0374. Bonferroni’s *post hoc* tests showed significance between placebo and nicotinamide riboside in CD157KO mice, *P* < 0.001. **(c)** Time spent in the dark zone. Two-way ANOVA; treatment, *F*_1,34_ = 5.363 *P* = 0.0267; interaction between genotype and treatment, *F*_1,34_ = 8.005, *P* = 0.0078). Bonferroni’s *post hoc* tests revealed significance between placebo and nicotinamide riboside in CD157KO mice, *P* < 0.01 (**d**) The average speed in the light compartment.Two-way ANOVA; for genotype (*F*_1,34_ = 4.593, *P* = 0.0393). *P < 0.05, **P < 0.01, and, ***P < 0.001, respectively.

Provision of daily NR dramatically relieved behavioural impairments in the light/dark test as measured by the number of entries (Fig. 3a; two-way ANOVA; *F*_1,34_ = 5.766, *P* = 0.022; genotype *F*_1,34_ = 2,871, *P* = 0.0993; interaction between genotype and treatment, *F*_1,34_ = 2.109, *P* = 0.1556). Bonferroni’s *post hoc* analysis revealed a significant difference between placebo and NR in CD157KO mice, P < 0.05. Latency to the first entry was significantly decreased by daily NR in CD157KO mice (Fig. 3b; two-way ANOVA; effect of genotype *F*_1,34_ = 3.106, *P* = 0.087; treatment, *F*_1,34_ = 12.62, *P* = 0.0011; genotype and treatment interaction, *F*_1,34_ = 4.695, *P* = 0.0374). Bonferroni’s *post hoc* tests showed a significant difference between placebo and NR in CD157KO mice, *P* < 0.001. Time spent in the hidden zone markedly increased in CD157KO mice treated with NR (Fig. 3c; two-way ANOVA; treatment, *F*_1,34_ = 5.363, *P* = 0.0267; interaction between genotype and treatment, *F*_1,34_ = 8.005, *P* = 0.0078). Bonferroni’s *post hoc* tests revealed a significant difference between placebo and NR in CD157KO mice, *P* < 0.01. No effects were found for average speed (Fig. 3d; two-way ANOVA; treatment *F*_1,34_ = 1.855, *P* = 0.1822; treatment between genotype interaction, *F*_1,34_ = 0.6007, *P* = 0.4437), except for a significant effect of genotype (*F*_1,34_ = 4.593, *P* = 0.0393). These data establish that CD157KO mice are capable of feeling the reward of being in the dark if they can overcome the inertia of being put in an uncomfortable place.

### NR does not correct anxiety-like behaviors in the open field test

**Figure 4:**
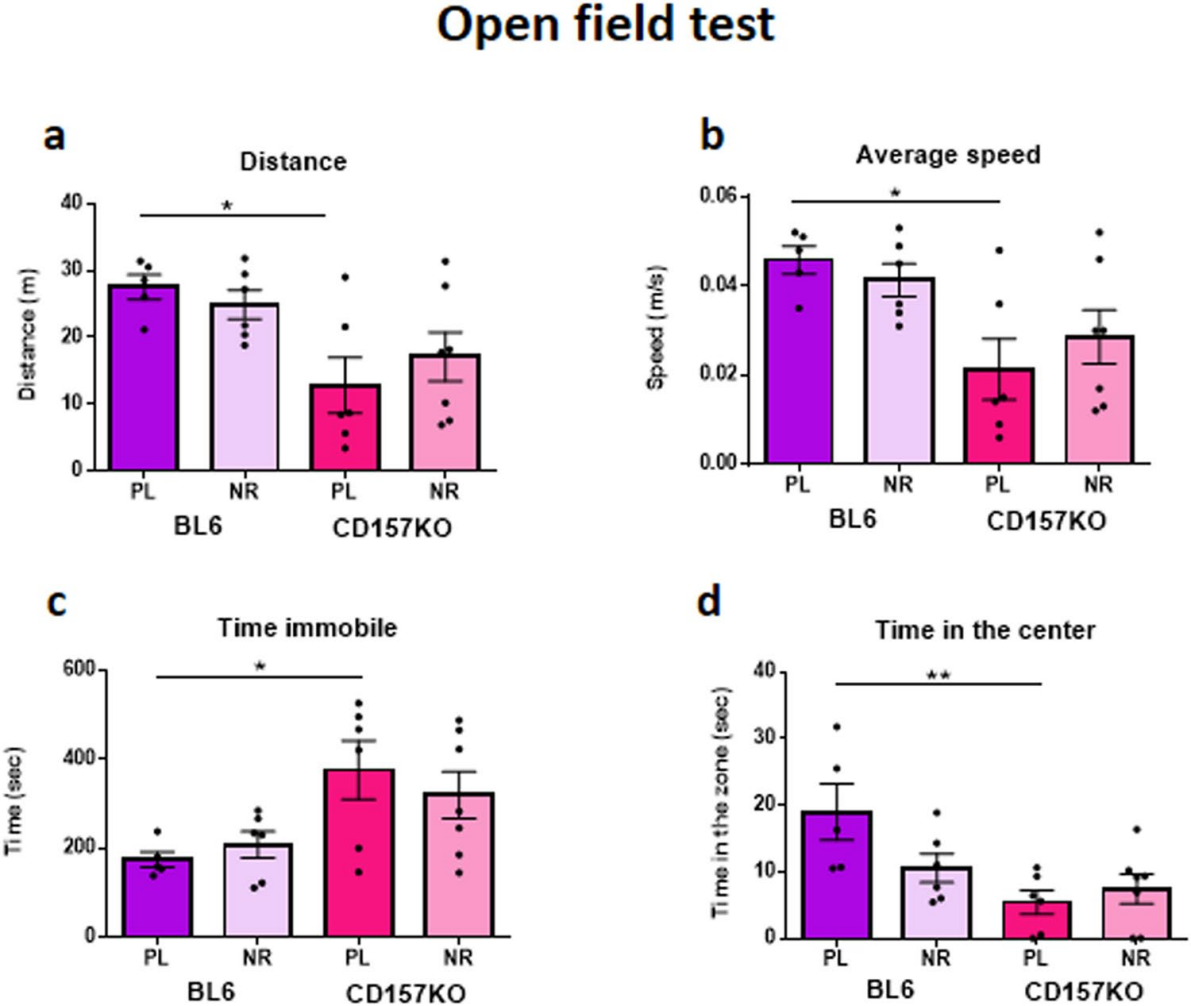
The behaviour of male mice for 10 min in the open field test. Daily gavage supplementation of nicotinamide riboside (NR, 3 mg/mouse) for 12 days induced no effect on any behavior by wild-type (BL6) nor the behavior deficits by CD157KO mice, compared with those of placebo treatment (PL). Two way ANOVA reveals significant effects only in the genotype: (**a**) distance traveled, *F*_1,20_ = 11.71, *P* = 0.0027; (**b**) average speed, *F*_1,20_ = 11.69, *P* = 0.0027; (**c**) time immobile, *F*_1,20_ = 10.38, *P* = 0.0043; (**d**) time spent in the centre of the open field *P < 0.05, and **P < 0.01.

In the open field, wild-type mice cover more distance at greater speed with less time immobile and more time in the centre than do CD157KO male mice. Daily NR did not rescue these behavioural deficits compared with the placebo treatment in CD157KO mice (Fig. 4; only the genotype effect was significant by two-way ANOVA; Fig. 4a; distance travelled, *F*_1,20_ = 11.71, *P* = 0.0027; Fig. 4b; average speed, *F*_1,20_ = 11.69, *P* = 0.0027; Fig. 4c; time immobile, *F*_1,20_ = 10.38, *P* = 0.0043; Fig. 4d; time spent in the centre of the open field arena, *F*_1,20_ = 10.32, *P* = 0.0044, no effect of treatment *F*_1,20_ = 1.506, *P* = 0.234). A trend toward significance was seen in the interaction between genotype and treatment, however *F*_1,20_ = 4.059, *P* = 0.0576.

### Social preference defecit and anxiety of CD157KO males are best corrected at a relatively low dose of NR

**Figure 5:**
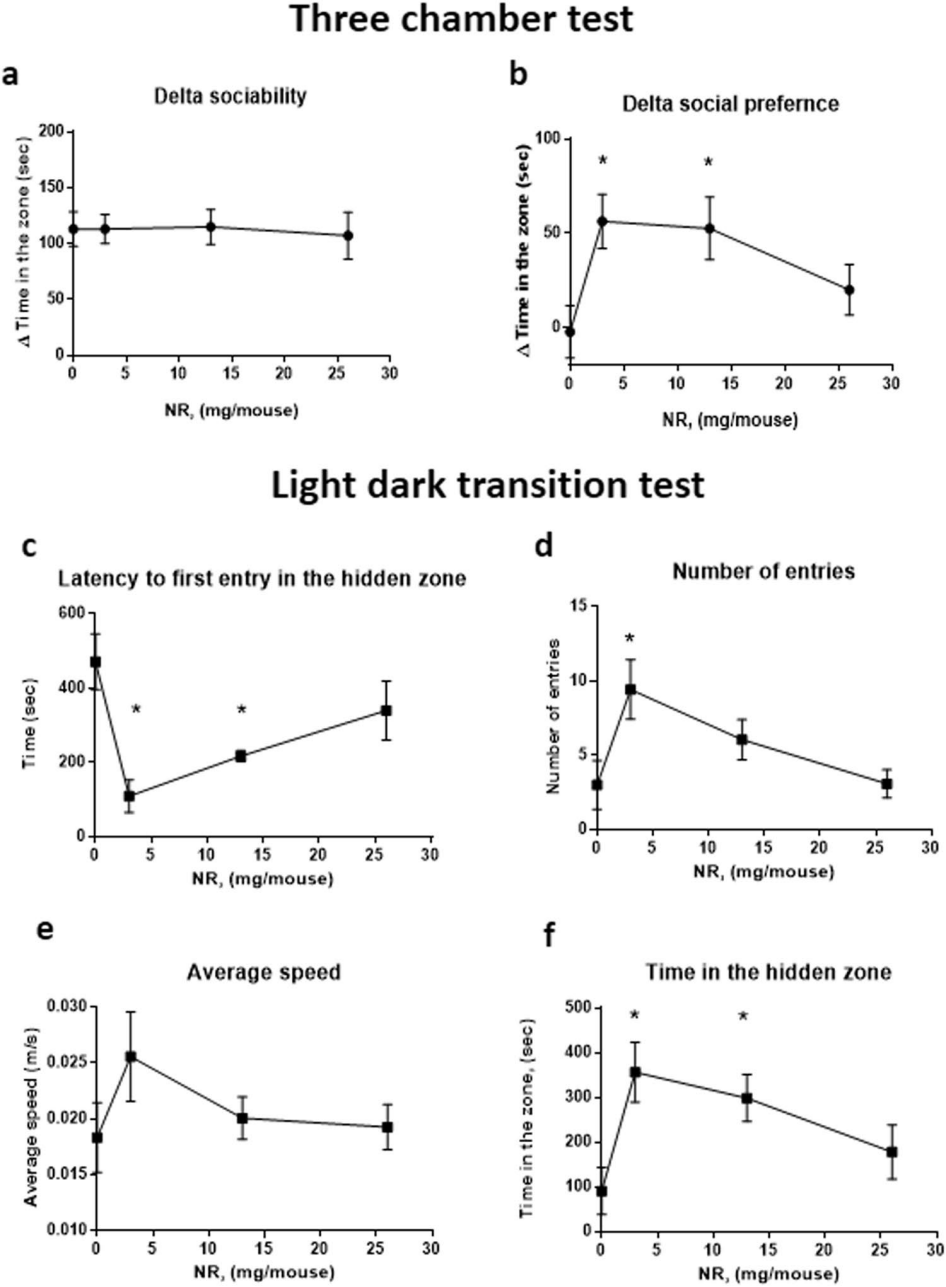
Effects of different doses of nicotinamide riboside (NR) on CD157KO mice in the three-chamber and light-dark transition tests. Different doses (3–26 mg/mouse) of gavage NR was administered for 12 days. Sociability (**a**) and social preference (**b**) are shown as time with the Stranger 1 subtracted by that with the non-social target or as time with Stranger 2 subtracted that of Stranger 1, respectively, in the three chamber test. One-way ANOVA followed by Bonferroni’s post hoc comparison: *F*_3,69_ = 3.873, *P* = 0.0128: *P < 0,05, from values with no NR. (**c**-**f**) Three **c**hamber box test. (**c**) Latency in first entry into the hidden (dark) compartment (One way ANOVA followed by Bonferroni’s *post hoc* comparison *F*_3,41_ = 3.698, *P* = 0.0191). (**d**) Transition numbers (One way ANOVA followed by Bonferroni’s *post hoc* comparison *F*_3,41_ = 3.272, *P* = 0.0306). Bonferroni’s *post hoc* comparison revealed *P < 0.05 compared with no nicotinamide riboside. (**e**). No dose-dependent **e**ffect was found in the average speed. (**f**) Time spent in the hidden zone (One way ANOVA followed by Bonferroni’s *post hoc* comparison *F*_3,41_ = 3.357, *P* = 0.0278). Bonferroni’s *post hoc* comparison revealed *P < 0.05 compared without NR.

We examined the dose-dependence of oral NR on the restoration of social behavioural impairments observed in the three-chamber test (Fig. 5a,b) and light/dark box (Fig. 5c–f). Each of the following four metrics was the most substantially ameliorated at 3 mg per day: Fig. 5b, delta social preference to new mice (one-way ANOVA, *F*_3, 69_ = 3.873, *P* = 0.0128); Fig. 5c, latency of the first entry into the hidden (dark) compartment (one-way ANOVA, *F*_3,41_ = 3.698, *P* = 0.0191); Fig. 5d, number of transitions into the dark zone (one-way ANOVA, *F*_3,41_ = 3.272, *P* = 0.0306); and Fig. 5f, time spent in the hidden (dark) zone (one-way ANOVA, *F*_3,41_ = 3.357, *P* = 0.0278). However, no dose effect was detected for delta sociability (time to social target; Fig. 5a; one-way ANOVA, *F*_3,69_ = 0.03477, *P* = 0.9912) or average speed of locomotion in the light zone (Fig. 5e; one-way ANOVA, *F*_3,41_ = 1.142, *P* = 0.3435). Beneficial effects were still seen at 13 mg per day (P < 0.05 in social preference (Fig. 5b), latency (Fig. 5c) and time in the dark (Fig. 5f) but were nearly eliminated at the highest dose of 26 mg/day (Fig. 5b–f).

### NR elevates brain NAD^+^ and cerebrospinal OT

**Figure 6:**
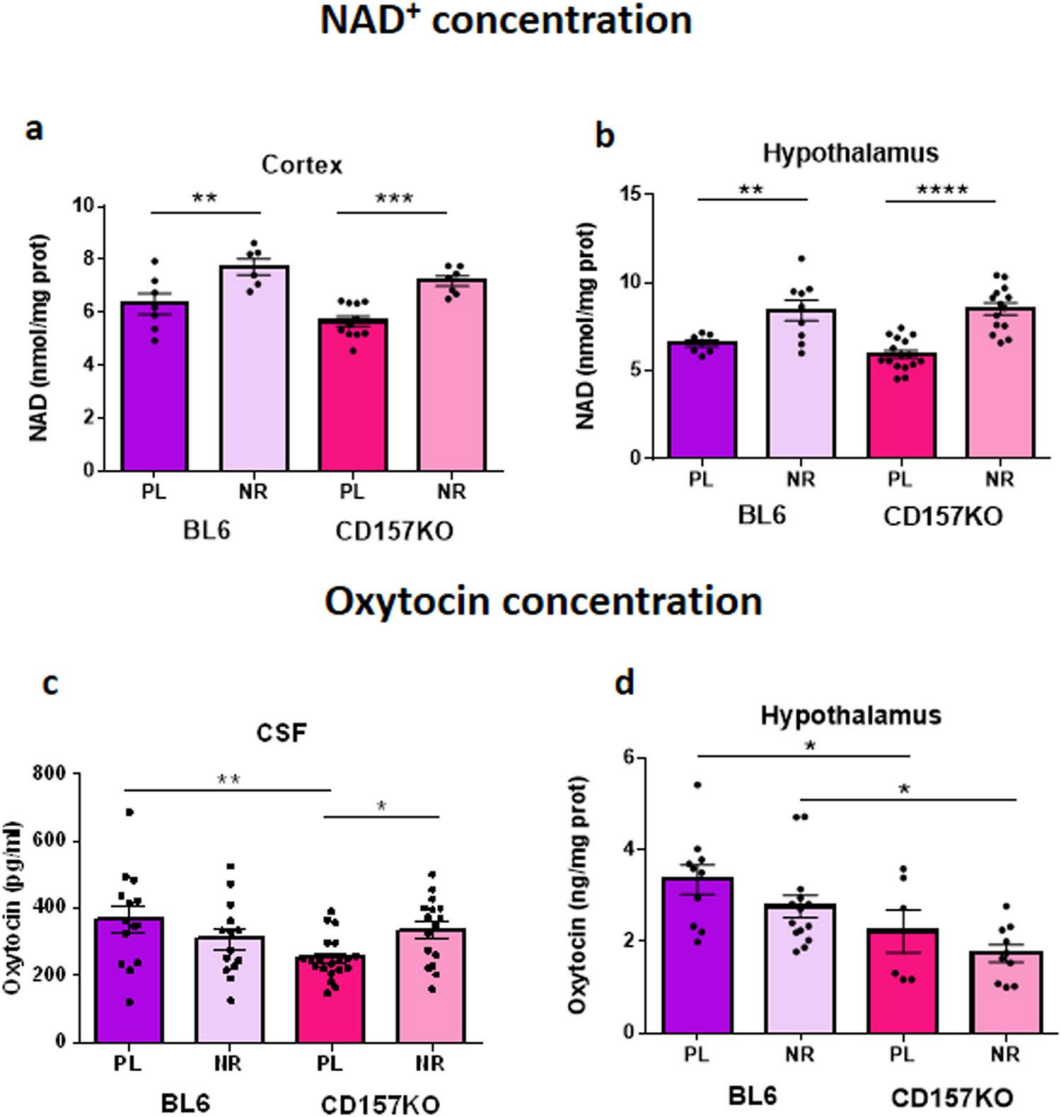
NAD^+^ and OT levels were determined in the cortex and hypothalamus or cerebrospinal fluid (CSF) of wild-type (BL6) and CD157KO mice treated with NR. (**a,b**) NR gavage at 3 mg/mouse for 12 days significantly elevated NAD^+^ concentrations in mice of both genotypes in the cortex (two-way ANOVA; the effect of treatment (*F*_1,27_ = 28.33, *P* < 0.0001) and the effect of genotype (*F*_1,27_ = 4.74, *P* = 0.0384) and the hypothalamus (two-way ANOVA; the effect of treatment (*F*_1,42_ = 36.58, *P* < 0.0001). Bonferroni’s *post hoc* comparison shows a significant difference between placebo and NR in CD157KO mice (*P* < 0.05)), comparing saline treatment (PL). (**c**) The identical treatment of NR significantly increased OT concentrations in the CSF of CD157KO mice (Two-way ANOVA revealed a significant effect of interaction between treatment and genotypes (*F*_1,60_ = 7.341, *P* = 0.0088). Bonferroni’s *post hoc* comparison shows a significant difference between placebo and NR in CD157KO mice (*P* < 0.05). (**d**) OT concentrations in the hypothalamus in wild-type and CD157KO mice showed no significant difference after treatment with NR (Two-way ANOVA showed the only effect of genotype (*F*_1,36_ = 12.59, *P* = 0.0011). *P < 0.05, **P < 0.01, ***P < 0.001, and ****P < 0.0001, respectively.

NAD^+^ levels were determined in the cortex and hypothalamus of mice treated with NR. As shown in Fig. 6a,b, both wild-type and CD157KO mice showed that oral NR increased NAD^+^ in both brain regions. In the cortex, two-way ANOVA revealed an effect of treatment (*F*_1,27_ = 28.33, *P* < 0.0001) and genotype (*F*_1,27_ = 4.74, *P* = 0.0384), but there was no interaction between them (*F*_1,27_ = 0.05429, *P* = 0.8175). In hypothalamic tissues, two-way ANOVA indicated an effect of treatment (*F*_1,42_ = 36.58, *P* < 0.0001) but no effect of genotype (*F*_1,42_ = 0.5062, *P* = 0.4807) or interaction (*F*_1,42_ = 0.9283, *P* = 0.3408).

OT plays important roles in sociability and the ability to overcome irrational fears^33^. Moreover, cADPR formation has been linked to OT release^11,12,15^, and OT administration has been used to correct ASD-like phenotypes in CD157KO mice^13,29^. To test the hypothesis that the NAD^+^-elevating and ASD phenotype-reverting effects of NR are accompanied by increased OT circulation, we assayed the levels of OT in the CSF and hypothalamus in male wild-type and CD157KO mice (Fig. 6c,d). As shown in Fig. 6c, CD157KO mice have depressed CSF OT levels that are reversed by oral NR. Two-way ANOVA revealed no effect of treatment (*F*_1,60_ = 0.2141, *P* = 0.6453) or genotype (*F*_1,60_ = 2.865, *P* = 0.0957) but a significant effect of their interaction (*F*_1,60_ = 7.341, *P* = 0.0088). Bonferroni’s *post hoc* comparison shows a significant difference between placebo and NR in CD157KO mice (*P* < 0.05). However, no increase in OT concentrations in the hypothalamus was observed (Fig. 6D). Two-way ANOVA showed an effect of genotype (*F*_1,36_ = 12.59, *P* = 0.0011) but no effect of treatment (*F*_1,36_ = 3.115, *P* = 0.0861) or their interaction (*F*_1,36_ = 0.02442, *P* = 0.8767).

## Discussion

The results demonstrated that the daily oral administration of NR rescued the social behavioural impairments observed in male CD157KO mice. NR had essentially no effects on social behaviour in wild-type male mice. The beneficial effects of NR appear to depend on restoration of CSF OT levels because the NR-induced OT elevation was only detected in CD157KO mice, which have a CSF OT defecit.

Beneficial effects of NR were not observed in female KO mice using the same parameters as those used to measure male behaviour in the three-chambered box test. More precisely, because there were no clear differences in behavioural impairments between CD157KO female mice and wild-type mice, we failed to detect NR effects in the current experimental conditions. It remains important to test NR using other parameters that enable the measurement of social behavioural impairments in female mice.

In the course of identifying a nutritional intervention for CD157KO mice, we reproduced the anxiety-like and social-avoidance-like deficits reported previously^13,30^. Reproducibly lower levels of CSF OT in male CD157KO mice make these mice an attractive model of autism, anxiety disorder, or social avoidance in neurodegenerative diseases. Significantly, this model responds to both OT and NR as a treatment.

The challenge of polygenic diseases of incomplete penetrance is that they are difficult to understand mechanistically. Multiple genetic and environmental (biochemical) factors may converge to dysregulate pathways that are altered in common conditions such as ASD^34^. We note that one potentially hopeful point when studying polygenetic diseases is that brain systems are redundant, and thus, it may be possible to increase normal functions that are only partially encoded by genetically damaged circuitry.

Our prior work implicated CD38 and CD157 in mediating calcium-dependent production of OT in social and anti-anxiolytic behaviours^8,12,15,29^. Therefore, we aimed to test whether the phenotypes of CD157KO mice might demonstrate reversibility in a pathway that depends on the formation of NAD, cyclic ADP-ribose, and OT. The data shown in this study clearly indicate that CD157KO males have depressed OT levels^13^, impaired social preference and a fear of exploring new places and that each of these phenotypes was reversed by oral NR, an agent that we showed elevated the brain NAD metabolome. While these deficits were induced by a loss of CD157, the beneficial effects of NR, therefore, cannot be conferred by CD157 activity. Our data suggest that increased NAD^+^ allows a higher level of CD38 activity to restore OT release, which allows CD157KO mice to reveal their innate ability to recognize and interact with a stranger and to move from the light to a darker, more comfortable place.

NAD^+^ is consumed by CD38 in formation of cyclic ADP-ribose^6,7^. It then participates in OT release in the hypothalamus^11,15^. In our study, ADP-ribosyl cyclase activity was maintained at a similar range as that in wild-type animals (data not shown). A recent study suggested that NR supplementation did not change CD38 expression^35^. However, *in vitro* studies have shown that NAD^+^ applied to the mouse hypothalamus leads to OT release^36^. It is reasonable to assume that an elevation in NAD^+^ levels by NR in the hypothalamus is responsible for repair of the OT release.

Our results also show that increased NAD^+^ levels repair OT production in CD157KO mice that restores their innate ability to recognize and interact with a stranger or to enter a new environment (dark)^30^. In the experimental neurosciences, social recognition is usually defined as an interest in novel social objects. Social memory is defined as a decrease in investigative behaviours observed in a rodent re-exposed to a familiar conspecific^37,38^. Among the various neurotransmitters involved in social recognition, OT is reported to be involved in social interaction, social recognition, and memory in the social brain^8,12^. The disruption of the OT system impairs social recognition and mutual interactions in humans with psychiatric disorders, such as ASD^39,40^; or schizophrenia^41,42^. Anxiety-, depression-, avoidance-, and hyperactivity-like behaviours in rodents are considered psychiatric disorder model phenotypes^13,43^.

Future work will probe CD38 dependence and the cell-type dependence of the beneficial effects of NR on CD157KO behaviour, the potential benefits of NR in other ASD models, and the potential of NR to become a safe nutritional intervention, in addition to OT^40,44^, for at least some types of ASD in human populations.

## Methods

### Animals

*Cd157/Bst1*^*−/−*^ (CD157KO of the C57BL/6 background) mice were as described previously^15,45^. CD157KO mice were maintained by crossbreeding homozygous mutant mice. Most experiments were performed using the congenic based method on selected adult males and females of the homozygous KO groups. C57BL6/N (8 weeks old, 23–27 g body weight) mice were obtained from Japan SLC Inc. (Hamamatsu, Japan) via a local distributor (Sankyo Laboratory Service Corporation, Toyama, Japan) and used as controls for the CD157KO mice. Half of the offspring of the wild-type mice and all the KO mice were bred in our laboratory colony, weaned at 21–32 days of age, and housed in same-sex groups of 5 sibling pairs that were kept in 1 cage in the animal center under standard conditions (24 °C; 12/12-h light/dark cycle, with lights on at 8:45 a.m.) with food and water *ad libitum*. After the pretest and during tests each mouse was housed in an individual cage.

All animal experiments were carried out in accordance with the Fundamental Guidelines for Proper Conduct of Animal Experiments and Related Activities in Academic Research Institutions under the jurisdiction of the Ministry of Education, Culture, Sports, Science and Technology of Japan and were approved by the Kanazawa University Committee on Animal Experimentation.

### Animal treatment

Mice were treated with NR (ChromaDex, Irvine, CA, USA) dissolved in physiological saline (PBS) by gavage in the doses of 3–26 mg in 100 μL solution or equivalent volume of PBS as placebo control. Mice were treated daily over 12 days.

### Social behaviour test in three-chamber boxes

The preference test for social targets of mice was performed using a three-chamber box. (Fig. 2). The apparatus was a rectangular, three-chambered box covered with clear polycarbonate. Dividing walls had doorways allowing access into each chamber. At the end of each test, the apparatus was sprayed with 1% sodium hypochlorite and 70% ethanol and wiped clean with paper towels^13^. The following procedure was used for the social behaviour test:

1. *Habituation*. The day before the test mice were habituated in an empty apparatus for 20 minutes. Age and sex matched target mice were also habituated for 20 min in small cages. On the day of the experiment the test mouse was first placed in the middle chamber and allowed to explore for 5 min with free access to all parts of the arena. Each of the two sides contained an empty wire cage (70 mm × 90 mm × 70 mm and bars spaced 5 mm apart). Zones placed at 2.5 cm intervals around the wire cages were assigned as zones of direct interaction (cage zone)
2. *Sociability*. After habituation, an unfamiliar mouse (Stranger 1; a naïve C57BL/6 male) was placed in the wire cage (in the left chamber); the other wire cage (in the right chamber) was left empty, and the test mouse was placed in the centre compartment of the social test box and allowed to explore for a 5-min session, with free access into the two side chambers. The amount of time spent in the cage zone in each chamber was measured using a digital video system and ANY-maze software (Stoelting Co., Wood Dale, IL, USA).
3. At the end of the 5-min sociability test, each mouse was further tested in a third 5-min session to quantitate preference for spending time with a new stranger. The new unfamiliar mouse (Stranger 2; an experiment naïve C57BL/6 N male) was placed in the wire cage (in the right chamber) that was empty during the previous 5-min session. The test mouse had a choice between the first, already-investigated, now-familiar mouse (Stranger 1) and the novel unfamiliar mouse (Stranger 2).

As described above, the amount of time spent in each chamber and in the direct interaction zones was measured using a digital video system and ANY-maze software. At the end of each test, the three-chamber box was cleaned as described above. The mean time interval between sessions was 2–3 min.

### Light-dark transition test

Anxiolytic-like or anxiogenic-like activity in mice was examined by the light-dark transition test^13,31^. The test box (200 mm × 600 mm × 200 mm) consists of a small dark safe compartment (one-third, 200 mm × 200 mm × 200 mm – *dark box*) and a large illuminated aversive compartment (two-thirds, 400 mm × 200 mm × 200 mm – *light box*). Each mouse was placed in the center of the light chamber without prior training. The mouse was allowed to run freely between the two chambers for 10 min. The trial was recorded using the ANY-maze video system. Latency to first entry (defined by all four paws entering), number of entries, time spent in the dark zone, and average speed in the light chamber were recorded.

### Open field test

The open field test was performed as described previously^13,30^. Briefly, the open field chamber consisted of a square wooden box (550 × 600 × 400 mm), with the inner surfaces covered with polypropylene sheets. The open field was divided into a centre zone (160 × 160 mm) and periphery. First, a mouse was placed in the arena for 10 min (session 1), then returned to its home cage. The time spent in the centre zone, total distance traveled, and immobility time were analyzed using a digital video system and ANY-maze video tracking software. At the end of session 3, the test chambers were sprayed with 1% sodium hypochlorite and 70% ethanol and cleaned with paper towels. The time interval between sessions was 2–3 min.

### Oxytocin measurement

Mice cerebrospinal fluid (CSF) was collected as described by Jin *et al*.^11^. Hypothalamus tissue was homogenized in 20 × volume of PBS. Homogenate was mixed with equal volume 0.4 M acetic acid and then centrifuged at 1000 rpm for 15 min. Supernatant was used for oxytocin measurement.

An oxytocin ELISA kit (Enzo Life Sciences, Farmingdale, NY, USA) was used for the oxytocin level determination^29^.

Protein levels were measured using a Bio-Rad protein assay kit (Bio‐Rad, Hercules, CA, USA).

### NAD measurement

β-NAD^+^ levels were measured using the enzymatic method^46,47^. Hypothalamus samples from the control and treated groups were harvested. The tissues were weighted and sonicated for homogenization in extraction buffer (50 mM potassium phosphate buffer containing 100 mM nicotinamide). Homogenate was incubated at 90 **°**C for 90 sec and then centrifuged at 8,000 x g for 3 min. The supernatant was incubated in reaction mix (32.5 mM glycylglycine, 50 mM nicotinamide, 0.25 M ethanol, 0.083 mg/mL MTT, 0.27 mg/mL (Phenazine Methosulfate, PMS), and the reaction was started by adding alcohol dehydrogenase (12.5 IU/mL). Increase in optic density at 570 nm was registered by a Multiscan GO Microplate Spectrophotometer (Thermo Fisher Scientific, Waltham, MA, USA). NAD levels were normalized to protein (milligrams of tissue). Protein levels were measured with a Bio-Rad protein assay kit (BioRad, Hercules, CA, USA).

### Statistical analysis

The data are expressed as the means ± SEM. The comparisons were evaluated using Student’s *t*-test, and One-way or two-way ANOVA, followed by post hoc Bonferroni test. In all analyses, *P* < 0.05 indicated statistical significance.

## Supplementary information

Supplementary.
