# Supplementary material for: Nicotinamide riboside supplementation corrects deficits in oxytocin, sociability and anxiety of CD157 mutants in a mouse model of autism spectrum disorder
Source: Sci Rep. 2020 Jun 22;10:10035. doi: 10.1038/s41598-019-57236-7 (PMC7308284; doi:10.1038/s41598-019-57236-7)
Supplement: Supplementary file 1 — Supplementary. [file 41598_2019_57236_MOESM1_ESM.doc]

**Nicotinamide riboside supplementation corrects deficits in oxytocin, sociability and anxiety of CD157 mutants in a mouse model of autism spectrum disorder**

Maria Gerasimenko^1,2^, Stanislav M. Cherepanov^1^, Kazumi Furuhara^1^, Olga Lopatina^1,3^, Alla B. Salmina^1,3^, [Anna A. Shabalova](http://www.mdpi.com/search?authors=Anna A. Shabalova&orcid=)^1,2^, ChiharuTsuji^1^, Shigeru Yokoyama^1^, Katsuhiko Ishihara^4^, Charles Brenner^5^, Haruhiro Higashida^1,2,3^

*^1^Department of Basic Research on Social Recognition and Memory, Research Center for Child Mental Development, Kanazawa University, Kanazawa 920-8640, Japan*

*^2^ Department of Socioneurosciences, United Graduate School of Child Development, Osaka University, Kanazawa University, Hamamatsu University School of Medicine, Chiba University, and University of Fukui, Kanazawa Campus, Kanazawa 920-8640, Japan*

*^3^Laboratory for Social Brain Studies, Research Institute of Molecular Medicine and Pathobiochemistry, and Department of Biochemistry, Krasnoyarsk State Medical University named after Prof. V. F. Voino-Yasenetsky, Krasnoyarsk 660022, Russia*

*^4^Department of Immunology and Molecular Genetics, Kawasaki Medical School, Kurashiki, Okayama 701-0192, Japan*

*^5^Department of Biochemistry, Carver College of Medicine, University of Iowa, Iowa City, IA 52242, USA*

Correspondence: charles-brenner@uiowa.edu and [haruhiro@med.kanazawa-u.ac.jp](mailto:haruhiro@med.kanazawa-u.ac.jp)


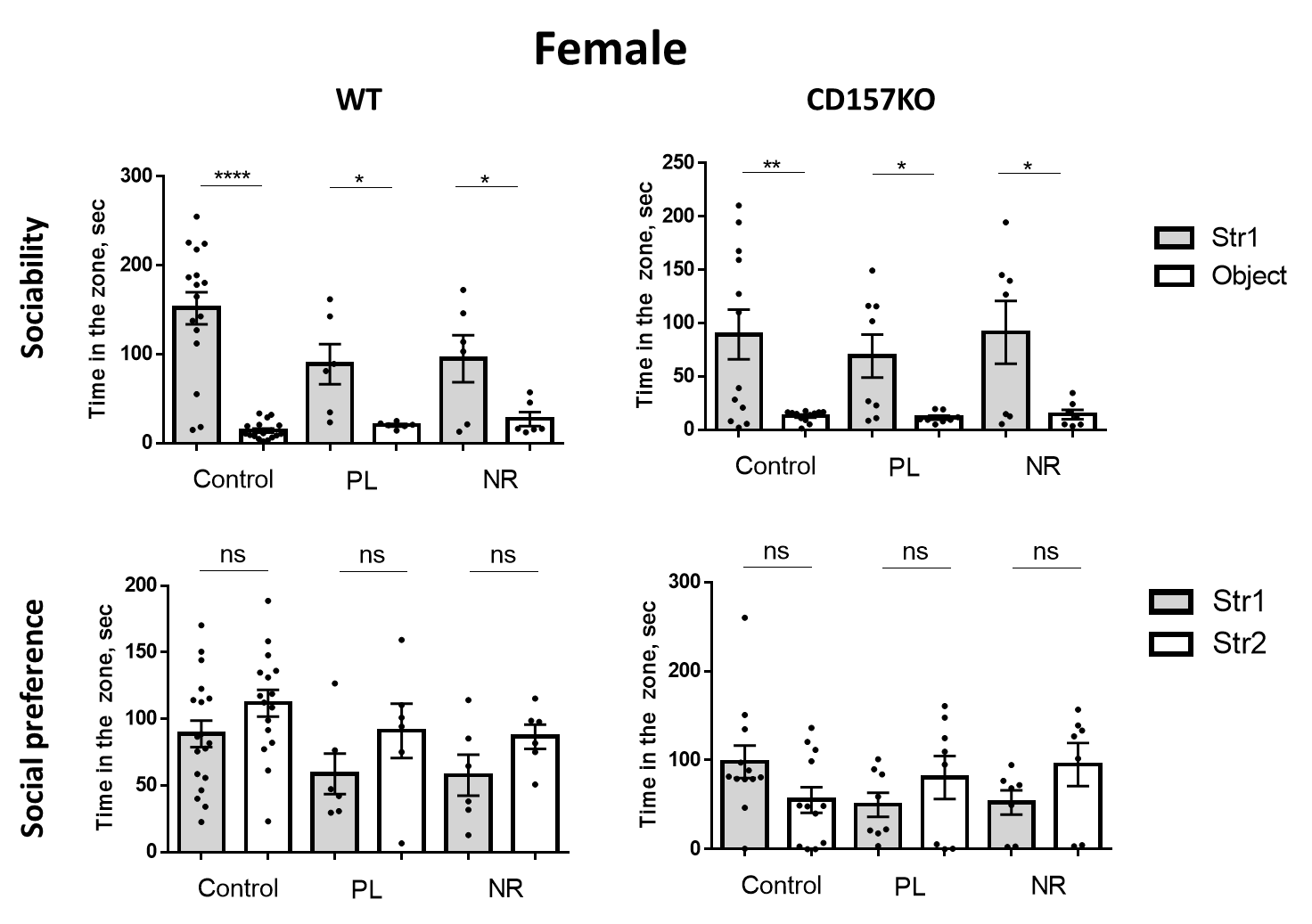


**a**

**b**

**c**

**d**

**Supplementary Figure 1.** **Social interaction observed in adult female mice treated with or without placebo (PL) or nicotinamide riboside (NR) in the three-chamber box test. (a)** The sociability stage in WT mice. **(b)** The sociability stage in CD157KO mice. **(c)** The social preference in WT mice. **(d)** The social preference in CD157KO mice. **p* < 0.05; ***p* < 0.01; *****p* < 0.0001.
